# P06-08 Socio-demographic profile of physically inactive adults living in Italy according to the PASSI data

**DOI:** 10.1093/eurpub/ckac095.093

**Published:** 2022-08-29

**Authors:** Valentina Minardi, Valentina Possenti, Rosaria Gallo, Benedetta Contoli, Susanna Lana, Maria Masocco

**Affiliations:** 1 National Centre for Disease Prevention and Health Promotion, Istituto Superiore di Sanità, Rome, Italy; 2 Department of Prevention, Local Health Unit Turin City, Turin, Italy

**Keywords:** Behavioural risk factors, surveillance system, public health

## Abstract

**Background:**

Insufficient physical activity (PA) or physical inactivity (PI) is one of the ten leading risk factors for global mortality. PI leads to 20-30% increased risk of all-cause mortality and monitoring its current levels and trends in general population is essential to track progress towards health targets, identify at-risk groups, assess policies' effectiveness, guide future planning.

**Methods:**

PASSI (Progressi delle Aziende Sanitarie per la Salute in Italia - Progresses in ASSessing adult population health in Italy) is an ongoing cross-sectional Behavioral Risk Factor Surveillance System (BRFSS) that monitors prevalence and temporal trends for the major modifiable health-related risk factors in the adults (18-69 years) residing in Italy. Data are collected in the Local Health Units (LHU) by trained personnel who administer a standardized questionnaire telephonically to sampled people. In the period 2015-2018, 132,717 people were interviewed in more of 90% LHUs (89 out of 101 in 2018), achieving a response rate above 80%. Concerning PA/PI, respondents are classified as per the WHO indicators in: (i) Active - basing on levels achieved in leisure time and/or heavy work; (ii) Partially active - in leisure time and/or moderate work or in spare time and without regular work; (iii) Inactive - in leisure time with sedentary work or in spare time and without regular work. PASSI calculates prevalence of PA/PI overall and by socio-demographic characteristics, including 95% confidence intervals (CI), and a logistic regression model estimates adjusted prevalence ratios (APR).

**Results:**

In Italy, 28.8% (CI95%:28.5-29.1%) of adult population is featured by a sedentary lifestyle: PI is greater among women (32.4% *vs.* 25.1%; APR: 1.26), grows with age (34% in over50 *vs.* 24.7% among 18-34 year-old; APR: 1.34), is higher among deprived people both per economic difficulties (41.7% if many *vs.* 22.4% if none; APR: 1.39) and for education level (23.7% university *vs.* 47.2% primary/any; APR: 1.33). We observed a highly evident geographic gradient: PI amounts to 18.3% in the North, 25.2% in the Centre and 40.6% in the South. A multivariate analysis confirms these values are statistically significant.

**Conclusions:**

PASSI data provide strong evidence to support community prevention interventions on territorial planning or advice by health professionals.

